# Energy-conversion efficiency for producing oxy-hydrogen gas using a simple generator based on water electrolysis

**DOI:** 10.1038/s41598-024-73534-1

**Published:** 2024-10-25

**Authors:** Ahmed M. Mousa, Hassan A. A. Sayed, Khaled A. M. Ali, Nabil S. Elkaoud, Wael A. E. Mahmoud

**Affiliations:** 1 Department of Agricultural Machinery and Power Engineering, Faculty of Agricultural Engineering, Al-Azhar University, Cairo, 11751 Egypt; 2 Department of Agricultural Machinery and Power Engineering, Faculty of Agricultural Engineering (Assiut Branch), Al-Azhar University, Assiut, 71524 Egypt

**Keywords:** Renewable energy, Hydrogen fuel, Oxy-hydrogen, HHO generator, Water electrolysis, Energy harvesting, Engineering

## Abstract

Producing hydrogen efficiently through water electrolysis could greatly reduce fossil fuel consumption. As well as this renewable energy source will also help combat global warming and boost economic investment opportunities. This paper studied some factors affecting the performance of oxy-hydrogen/hydroxy (HHO) gas generator, such as applied voltage (from 10.5 to 13.0 V) and electrolyte solution concentration (from 0.05 to 0.20 M), using a dry fuel cell based on the electrolyzing technique of water. The results revealed that the HHO gas production rate, power consumption, and temperature change of electrolyte solution increased significantly with increasing the tested applied voltage and electrolyte concentration. This study concluded that the optimum conditions for producing HHO gas ranged from 11.5 to 12.0 V for applied voltage and from 0.05 to 0.10 M for KOH concentration according to the lowest specific energy and highest HHO gas generator efficiency. Under the previous optimum conditions, the highest productivity, specific energy, and efficiency of the HHO gas generator were 343.9 cm^3^ min^−1^, 3.43 kW h m^−3^, and 53.79%, respectively, using 12.0 V for applied voltage and 0.10 M for electrolyte solution concentration. These findings provide an unambiguous direction for adjusting the operational factors (applied voltage and electrolyte concentration) for efficient HHO gas production and use in different applications. Furthermore, the required energy to operate the HHO gas generator can be obtained from renewable sources.

## Introduction

Fossil fuels still supplies about 82% of all energy consumed globally^1,2^. However, fossil fuels generate greenhouse gas emissions such as CO, CO2, SOx, NOx, and unburned hydrocarbons (HC), contributing to climate change (global warming) and negatively impacting human health and the environment. Furthermore, fossil fuels become expensive due to increasing demand and the depletion of their sources^3,4^. In order to reduce the negative effects of the combustion of fossil fuels, it has become necessary to utilize renewable and ecologically friendly energy sources. Oxy-hydrogen or hydroxy (HHO) gas is a promising alternative fuel that has several advantages over fossil fuels^5^. The advantages of HHO gas include its high flammability, more oxygen, fast burning rate, and zero carbon compared to fossil fuels^6^. HHO gas is an effective fuel that can be used in different applications such as internal combustion engines, cooking, heating, desalinating water, welding, and metal cutting, etc. So oxy-hydrogen gas can be employed as an alternative energy source for solving the shortage problem of petroleum fuel and reducing environmental pollution^7^.

Holladay et al.^8^ and Nnabuife et al.^9^ classified water-splitting methods into three types: electrolysis, thermolysis, and photoelectrolysis^10–12^. Renewable and non-renewable resources are used to produce hydrogen gas^9^. Electrolysers can also use renewable energy surplus to produce green hydrogen, enhancing the electrical grid’s stability. In addition, medium- to long-term energy storage can be achieved with hydrogen^13^. The main features of the water-splitting methods are shown in Table 1.

**Table 1:** A few characteristics of solar energy to hydrogen conversion methods according to Wang et al.^14^.

Methods	Thermochemical	Electrolysis	Photoelectrolysis	Photochemical
Reaction mechanism	Thermal spitting	Electric potential	Electric potential	Photon-activated electron
Form of energy input	Thermal	Electricity	Electricity	Photon
Solar energy-capturing device	No	No	Yes	Yes
External or internal energy supply	External	External	Internal	Internal
Basic components for engineering apparatus	More than 3 thermal reactors	2 electrodes and electrolyte	2 electrodes, electrolyte, and sunlight window	At least 1 sensitizer, at least 1 catalyst, and sunlight window
H_2_ and O_2_ produced separately or in mixture	Separately	Separately	Mix	Mix
Overall production efficiency	45%	30%	16%	10%
Suitable for large scale production or fueling stations	Large scale	Medium scale and fueling station	Fueling station	Fueling station
Additional hydrogen distribution network	Needed	Depends on production scale	Not necessarily	Not necessarily

Wang et al.^14^ found that the technology of water electrolysis powered by solar electricity is more mature than other techniques. Additionally, for small-scale hydrogen production in distributed facilities, certain methods, particularly water electrolysis, may be more cost-effective^8^.

The electrolysis technique refers to an electrolysis cell that can separate hydrogen (H_2_) and oxygen (O_2_) from water molecules using DC electricity^15^. Water electrolysis can produce HHO gas from dry or wet cells^16^. When comparing two types of HHO gas production cells (dry and wet cells), the dry cell is vastly superior to the wet cell, producing significantly more HHO gas under identical input conditions. For cell operation and maintenance, the dry cell is more dependable and suitable than its wet cell; its safety features are far more reassuring than those of the wet cell^17^. The dry cell is favorable because of its simplicity, easy manufacturing, and assembly. HHO dry cell is economical, made from locally available materials, and can be used with internal combustion engines supplying its required energy from the battery^2^. Furthermore, HHO gas can be applied as an additional fuel in a diesel or gasoline engine without any modifications or the need for a storage tank; the addition of HHO gas to a diesel engine led to increased torque output by 19.1% whereas the average values of specific fuel consumption, CO emission, HC emission was decreased by 14, 13.5, and 5% respectively^18^. The generator of HHO gas was designed, constructed, and consisted of 3 anodes (A), 3 cathodes (C), and 20 neutral (N) plates from SS grade of 316 L and investigated the effect of voltage, time, and electrolyte solution concentration; the authors reported that; increasing voltage, time and electrolyte solution concentration lead to increase the yield of hydroxy gas^4^.

Many types of electrolytes with different concentrations can be used for the production of HHO gas, such as potassium hydroxide (KOH), sodium chloride (NaCl), sodium hydroxide (NaOH), sulphuric acid (H_2_SO_4_), etc., are added to pure water for increasing the electrical conductivity^19^. KOH produces more HHO gas than NaOH because it has a higher stability and compatibility with metallic components^20^. Also, potassium hydroxide is the perfect electrolyte to increase the generation of the brown gas; furthermore, vertical orientation is ideal for plates at a gap of 2–3 mm between each plate^21^. El-Kassaby et al.^22^ reported that the highest production of HHO gas was 18 L h^−1^ using 2 neutral plates, 1 mm distance, and 6 g L^−1^ of KOH. Also, the thermal efficiency of gasoline engines was increased by 10%; consequently, fuel consumption decreased by 34%. As well as the CO, HC, and NOx values for exhaust gas emissions decreased to approximately 18, 14, and 15%, respectively. Alam and Pandey^15^ investigated the effect of DC current and voltage, electrolyte solution concentration, reaction temperature, and time on the HHO gas production rate. Their results indicated that the optimum production of HHO was obtained at 1 A current, 5 V potential, and 1 mol electrolytic concentration. Also, the increase in voltage, cell temperature, and electrolytic concentration improved hydroxy gas production by about 30–40% with a reduction in energy consumption by about 35%. Mustaqim & Maulana^23^ produced HHO gas by using different concentrations from NaOH (1, 2, 3, 4, and 5 M) as electrolyte and LaCoO_3_ (0, 0.025, 0.05, 0.33 and 0.67 wt%) as catalyst. They reported that; the optimum production of HHO occurs using 3 M of Sodium hydroxide, whereas; the addition of LaCoO_3_ as a catalyst led to a drop HHO gas production rate for all tested concentrations of catalyst. Whereas Almassri et al.^24^ studied the effects of the consumed power, and reaction time on the production of HHO by using a dry HHO cell and a solution of ammonium hydroxide (NH_4_OH) as an electrolyte; they found that the productivity of HHO increases with increasing the consumed power and reaction time, for producing of one kilogram of hydrogen the energy consumption was about 70.5600 MJ. Also, they reported that the ammonium hydroxide induced significant corrosion of metals, which is considered a drawback of ammonia hydroxide.

Despite the availability of studies related to testing the performance of dry cell HHO gas generators using water electrolysis, there is still a gap in the literature when examining the performance indicators. Furthermore, little research has been conducted on the applied voltage effect on the performance of the hydroxy gas generator within the voltage range of rechargeable batteries (12 V) in addition to photovoltaic cell systems that produce power at the same voltage which may be used to run the hydroxy gas generator to produce the green hydrogen fuel. Also, few researchers have focused on developing hydroxy gas generators on a small scale for sustainable energy purposes. This highlights the need for more research to determine the optimal operating conditions to achieve the highest efficiency level.

Therefore, the main target of this research was to develop and manufacture an oxy-hydrogen gas generator from a dry cell type with high energy-conversion efficiency from locally available materials, characterized by simplicity in construction, ease of maintenance, and operation. Additionally, study some factors affecting the performance of HHO gas generator. Specifically, the impact of applied voltage and electrolyte solution concentration on productivity, power consumption, change in electrolyte temperature, specific energy requirements, and efficiency of HHO gas generator were studied using water electrolyzing under a laboratory scale.

## Materials and methods

### HHO gas generator

**Fig. 1:**
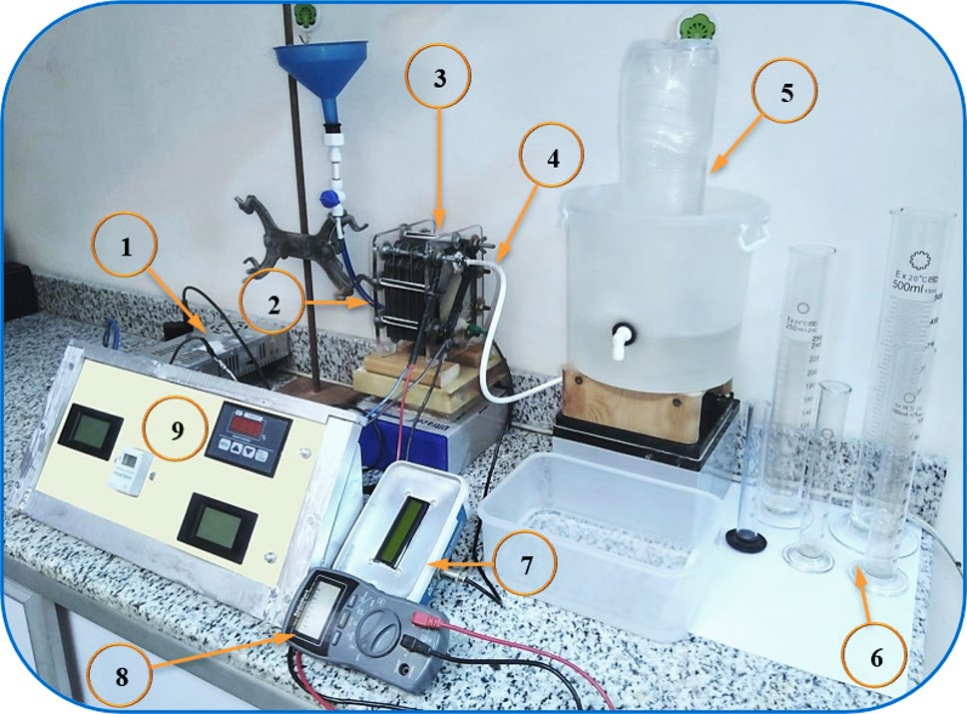
Final experimental system of HHO gas generator. (1) Power supply “DC”, (2) Electrolyte inlet, (3) Dry electrolyzer, (4) HHO outlet, (5) Bubbler, (6) Graduated cylinders, (7) Digital temperature gauge, (8) Digital AVO-meter and (9) Control and monitoring panel.

The developed HHO gas generator (Fig. 1) consists of five main parts used for oxy-hydrogen (HHO) gas production: power supply, control and monitoring panel, electrolyte temperature gauge, electrolyzer, and measuring tool of HHO volume.

#### Power supply

The power supply was used to convert AC (alternating current) into DC (direct current). The main specifications of the power supply are as follows: model number: S-360-12, AC input: 110/220 V ± 15%, DC output: 12 V 30 A, and contain adjustable range for DC voltage from 10.44 to 15.24 V.

#### Control and monitoring panel

A simple panel was carried out for control and monitoring. This panel consists of (1) AC digital multi-function meter; the product type is PZEM 061. This device can simultaneously measure and show electrical parameters (voltage, current, power, and energy) while operating the system. AC Voltage measurement range is 80 to 260 V with accuracy of 1 V. Current measurement range is 0 to 100 A with accuracy of 0.01 A. Power measurement range is from 0 to 22 kW with accuracy of 0.1 W in range (0.0 to 999.9 W), 1 W in range (1000 to 9999W) and 0.1 kW in range (10.0 to 22.0 kW). (2) A programmable switch timer from type TM-615 was used to set the operating time of the experiment; this timer was manufactured by Sinotimer and can be automatically switched on and switched off according to experiment time. (3) DC digital multi-function meter with a shunt (50A/75 mV); the type of product is PZEM 051. Also, this device can be measured and show electrical parameters (voltage, current, power, and energy) simultaneously while operating the system. DC Voltage measurement range is 6.5 to 100 V with an accuracy of 0.01 V. Current measurement range is 0 to 100 A with accuracy of 0.01 A. Power measurement range is from 0 to 10 kW with accuracy of 0.1 W in range (0.0 to 999.9 W), 1 W in range (1000 to 9999W). (4) Adjustable PWM (pulse width modulation) was used to restrict the amount of electric current flowing into the HHO generator. PWM is one of the techniques for controlling current and voltage via regulating the ratio of pulse width to the period of a square signal in the form of an applied periodic voltage to the electric load as a power source^25^. The main features of the PWM module used are as follows: Model: Q8-42S, input voltage: 6–60 VDC, output current: 0–30 A, and regulation range 0–100%.

#### Digital electrolyte temperature gauge

**Fig. 2:**
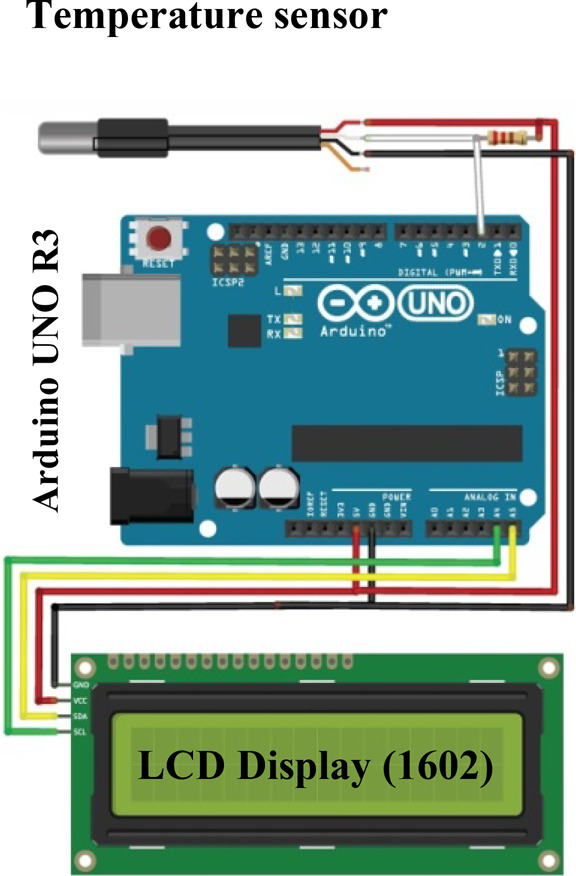
Electronic circuit diagram of electrolyte temperature measuring device.

The temperature gauge is an electronic circuit that consists of Arduino UNO R3 with microcontroller ATmega328 chip, waterproof temperature sensor, and Blue Screen LCD (1602) Display Module with IIC Interface for Arduino, as shown in Fig. 2. The temperature sensor was connected to the HHO cell to sense the temperature of the electrolyte solution.

#### Electrolyser

**Fig. 3:**
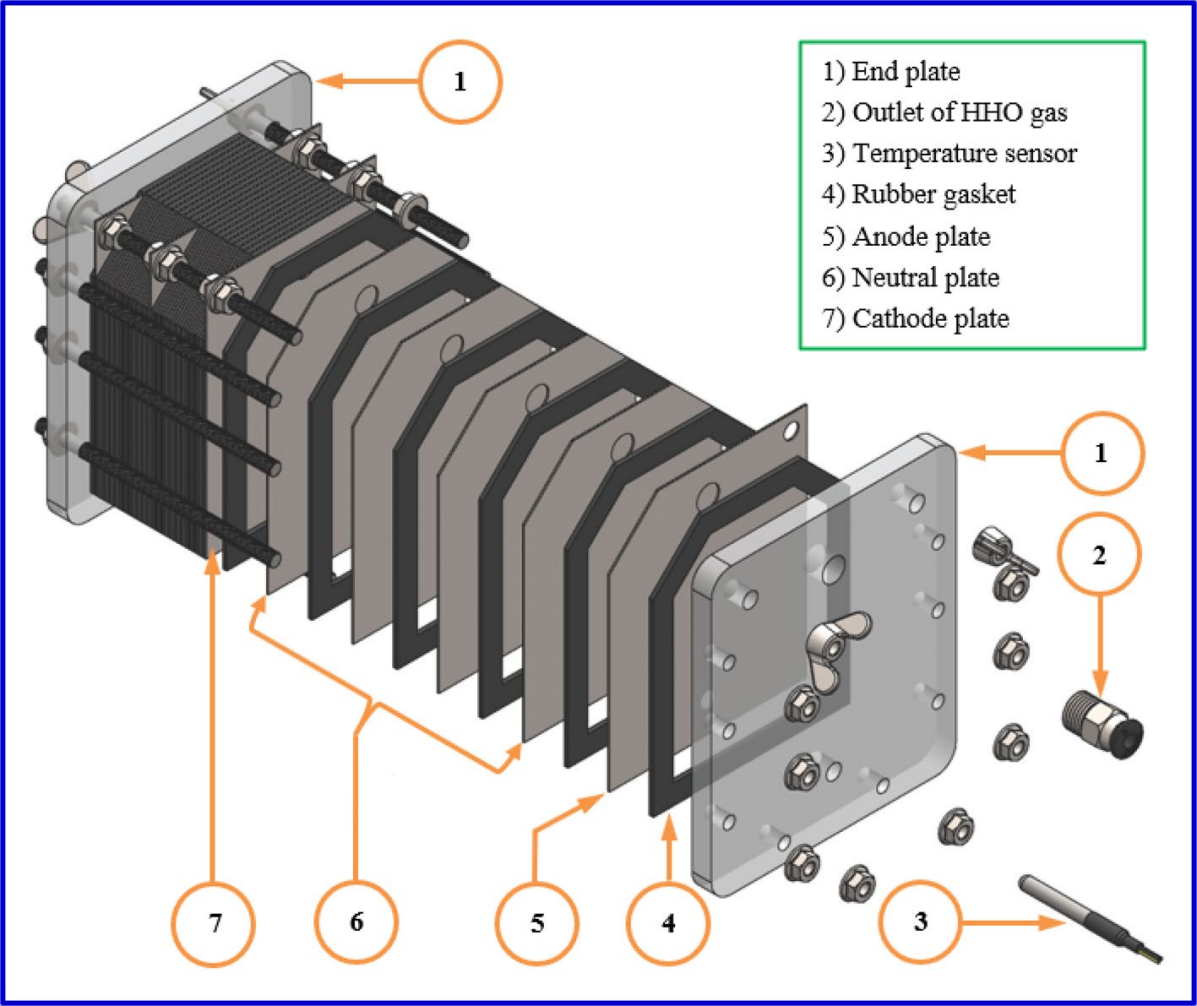
3D Assembly drawing of main body of the developed electrolyzer.

The developed electrolyzer is a type of dry fuel cell; this electrolyzer was designed and manufactured to produce HHO gas using a water electrolysis method. In this method, hydrogen and oxygen are separated by electrolysis of electrolyte solution. The mixture of produced gases (HHO) contains 66.6% hydrogen and 33.3% oxygen^26^. Distilled water is not electrically conductive, so potassium hydroxide (KOH) was added to distilled water to boost its electrical conductivity. The KOH was in the shape of scales, the minimum assay was 85%, the molecular mass was 56.11 g mol^−1^, and the KOH was purchased from EL-Nasr Pharmaceutical Chemicals Co. The main body of the electrolyzer (Fig. 3) comprises 26 electrodes, 27 rubber gaskets, and two plates of acrylic (Plexiglass). In addition, small installation components such as; fully threaded rods, screw nuts, hoses, pneumatic connectors, and electrical connection wires. The electrodes were made of 316-L grade stainless steel (SS). The SS material was chosen because it has high resistance to corrosion and rusting against alkaline solutions. The electrodes were arranged as follows: 1A 4N 1C and so on, where (A) represents the anode plate, (N) represents the neutral plate, and (C) represents the cathode plate. This arrangement was repeated to obtain the final electrode configuration of 3A 3C 20N. For a single stack, we used the following arrangement (1A 4N 1C = 6 plates = 5 cells). The voltage of each cell is computed by dividing the source voltage by the number of cells, resulting in 2.1 to 2.6 V. According to the applied voltage range (10.5 to 13 V) in our investigation, this agrees with Bob Boyce, “the approximate value of 2–3 V per cell allows the generator to operate at appropriate conditions”^7,27^. The main dimensions of the anode/cathode and neutral plates were 120 mm × 100 mm × 0.8 mm. The plates of the anode/cathode are cut from one side at the top, whereas the neutral plates are cut from the upper left and right sides. The goal of cutting both sides in the neutral plates is to avoid a direct connection between anode/cathode plates and neutral plates^28^.

Two holes were drilled in all electrodes (SS plates), the upper hole with a diameter of 11.5 mm to ensure exit of the HHO gas produced by the electrolysis process and the lower hole with a diameter of 7.5 mm to ensure evenly distributed electrolyte solution throughout all cell gaps. In addition, another hole was drilled in the anode/cathode plates at the top corner with a diameter of 7 mm to connect the electrical charge by using the two screw rods. A hollow rubber gasket with main dimensions of 120 × 100 × 2 mm was used to prevent leakage of the electrolyte solution and produced HHO gas, in addition to insulating the electrodes from each other. Two plates from transparent acrylic (Plexiglass) were used on the two sides of the electrolyzer as cover plates; this material is flexible and resistant to corrosion against corrosive materials. A hole was drilled in the bottom of the left acrylic plate for an electrolyte solution inlet. For the HHO gas outlet, a hole was drilled in the top of the right acrylic plate, as shown in Fig. 3. The general specifications of the developed HHO gas generator are shown in Table 2.

**Table 2:** Specifications of the developed Electrolyser.

Items	Details
HHO generator type	Dry cell
Plate material	Stainless steel (316 L)
The main dimensions of the plate, mm	120 × 120 × 0.8
No. of plates	26 plates
No. of rubber gaskets	27 gaskets
Thickness of gaskets, mm	2
Plate configuration	3A 3C 20N
Power input	12 V–DC
Catalyst electrolyte	KOH
Type of water	Distilled water

#### Measuring tool of HHO volume

The produced volume of HHO gas was measured by displacement water under atmospheric pressure. The displacement water compressed by HHO gas produced from the generator was collected and then poured into the graduated cylinder to determine its volume.

### The experimental procedure

The production of oxy-hydrogen/HHO gas was carried out using the electrolysis method of water according to the following steps: the electrolysis cell was washed with distilled water several times and then dried. For preparing the electrolyte solution with the required concentration, the required amount of potassium hydroxide was weighed by a digital electrical balance with an accuracy of 0.1 g and then dissolved manually in distilled water for about five minutes using a conical flask to obtain a homogeneous solvent. Then, the determined amount of electrolyte solution (315 cm^3^) was measured using a measuring cylinder and poured into the developed electrolysis cell. The electrolyte solution level is about 13 mm under the gas vent hole. The timer was set for the determined experiment time to 1 min for all experiments; before the electrolysis process began, the starting temperature of the electrolyte solution was constant at 32 ± 0.5 °C for all experiments. During the test, the power consumption in the electrolysis process was measured and recorded at the middle and finish of the experiment, and then the average value was taken. After the experiment time elapsed, the finish temperature of the electrolyte solution was measured and recorded. Then, the produced volume of HHO was measured using the displacement water method using graduated cylinders. After finishing and confirming that the HHO gas production process will run smoothly and without any problems, the following variables: applied voltage (10.5, 11.0, 11.5, 12.0, 12.5, and 13 V) and electrolyte concentration (0.05, 0.10, 0.15, and 0.20 M) were studied. All experiments were repeated five times, and the mean value was taken.

### Performance evaluation of the HHO gas generator

- Productivity of HHO gas generator

The productivity [${Q}_{g} ({\text{cm}}^{3}{\text{min}}^{-1})$] of HHO gas generator was calculated by using the following equation:

1 $$Q_{g} = \frac{{V_{HHO} }}{t}$$

where ${V}_{HHO}$ is the volume of HHO gas (${\text{cm}}^{3}$) and $t$ is the time of operating ($\text{min}$).

- Power consumption

The DC power consumption [*P* (W)] was directly measured by using the digital multi-function meter Model No; (PZEM-051); the measured value (DC power) while operating the HHO generator found that agreement with the value of calculated power via the equation shown below:

2 $$P = I \times V$$

where $I$ is the DC current consumed (Amperes) and $V$ is the DC voltage difference (Volts).

- Change in temperature

The change in electrolyte solution temperature *[*$\Delta T (^\circ \text{C})$] was calculated by using the following equation:

3 $$\Delta T = T_{F} - T_{S }$$

where ${T}_{F}$ is the final temperature ($^\circ \text{C}$) and ${T}_{S}$ is the starting temperature ($^\circ \text{C}$).

- Specific energy

The specific energy requirement [$ES (\text{kW h }{\text{m}}^{-3}$)] was calculated by using the following equation:

4 $$E_{s} = \frac{P}{{Q_{g} }}$$

where $P$ is the consumed power (kW) and ${Q}_{g}$ is the productivity of HHO gas $({\text{m}}^{3}{\text{h}}^{-1})$.

- Efficiency of HHO gas generator

The efficiency [$\eta (\text{\%})$] of the HHO gas generator is the ratio of energy gained and energy consumed^22^. The HHO gas is composed of two moles of hydrogen and one mole of oxygen, therefore the HHO gas composition of 1/3 volume percent oxygen and 2/3 percent hydrogen, so the volume of hydrogen is 0.66% of the produced volume of HHO gas (${V}_{{H}_{2}}=0.66 \times {V}_{HHO}$)^29^. The efficiency of the HHO gas generator was calculated as illustrated in the following formula^30^:

5 $$\eta = \frac{{V_{{H_{2} }} \times \rho_{{H_{2} }} \times LHV_{{H_{2} }} }}{P \times t} \times 100$$

where ${V}_{{H}_{2}}$ is the volume of hydrogen (${\text{m}}^{3}$), ${\rho }_{{H}_{2}}$ is the density of hydrogen ($0.0838\text{ kg }{\text{m}}^{-3}$), ${LHV}_{{H}_{2}}$ is the lower heating value of hydrogen ($120\times {10}^{6}\text{ J }{\text{kg}}^{-1}$), $P$ is the DC power consumption ($\text{W}$), and $t$ is the time ($\text{s}$).

The results obtained from this study were statistically analyzed using the following programs: Microsoft Excel and SPSS “V. 23”.

## Results and discussion

Performance of the developed HHO gas generator includes the following items: productivity, power consumption, amount of change in electrolyte solution temperature, specific energy requirement, and efficiency of HHO gas generator.

### Productivity

**Fig. 4:**
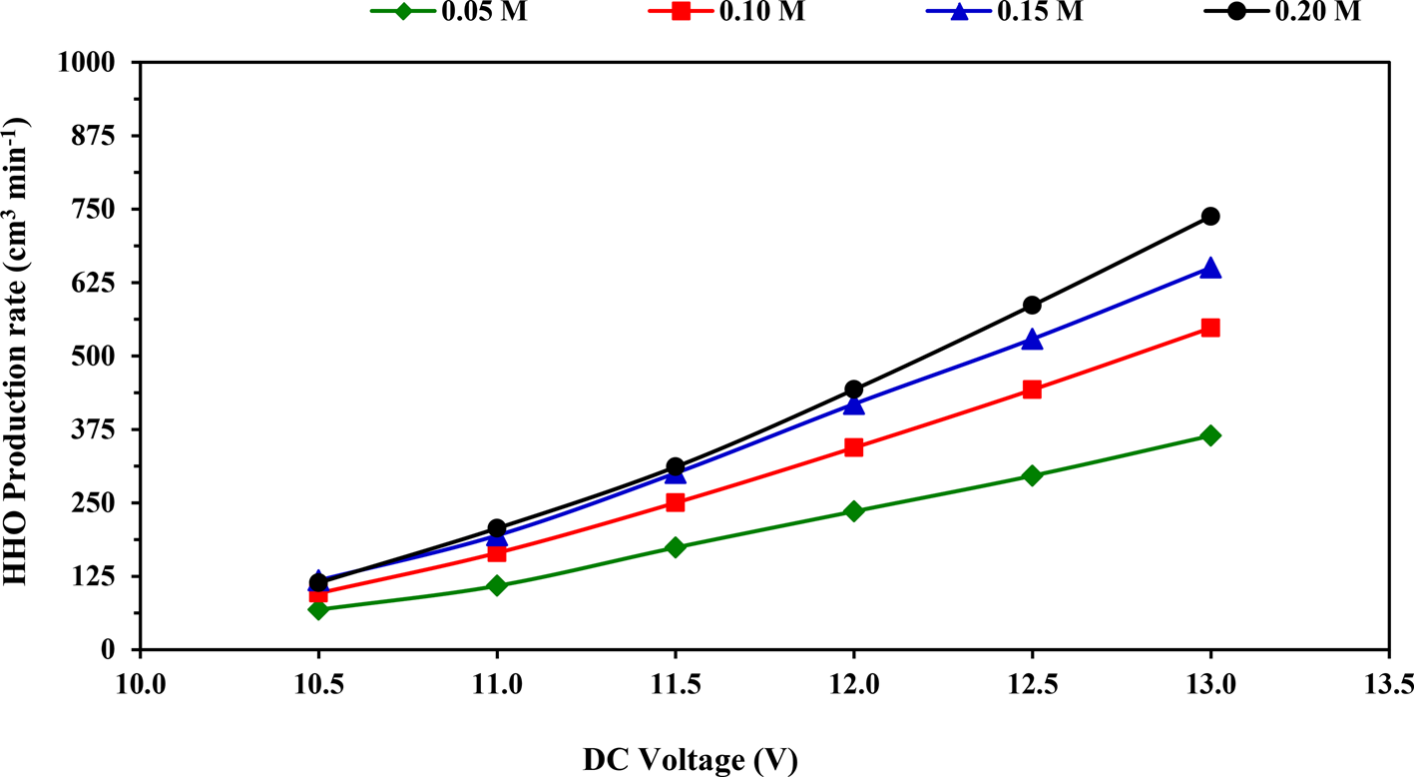
Impact of applied voltage on HHO gas production rate at tested KOH concentrations.

Figure 4 shows the impact of applied voltage on the rate of HHO gas production at tested KOH concentrations. As expected, it was found that the rate of HHO gas production increased gradually by increasing the applied voltage for all tested KOH concentrations, and a similar trend of this result was observed in the literature^4,15,16,31^. This result could be caused by an increase in the density of uniform charge, ions exchange on the surface of the electrode, and acceleration of the reaction kinetics^2^. The highest value of the HHO production rate is 737.6 cm^3^ min^−1^ at the applied voltage of 13 V using KOH concentration of 0.20 M, while the lowest value of the HHO production rate is 68.17 cm^3^ min^−1^ at applied voltage of 10.5 V using KOH concentration of 0.05 M. The rate of HHO gas production increased from 68.17 to 364.4, 96.25 to 547.8, 118.1 to 650.5, and 114.1 to 737.6 cm^3^ min^−1^ by increasing the applied voltage from 10.5 to 13.0 V at four tested KOH concentrations 0.05, 0.10, 0.15 and 0.20 M, respectively as displayed in Table 3.

**Table 3:** Average values of HHO production rate at tested voltages and electrolytic concentrations, [mean value ± (SD)] and Duncan’s test result.

DC voltage (V)	HHO Gas production rate (cm^3^ min^−1^)				Mean
	Electrolytic solution concentration (M)				
	0.05	0.10	0.15	0.20	
10.5	68.17 (3.06)	96.25 (2.42)	118.1 (2.82)	114.1 (4.82)	99.17^a^
11.0	108.8 (3.29)	164.9 (2.40)	194.7 (4.73)	206.8 (6.87)	168.8^b^
11.5	174.0 (8.15)	250.2 (12.0)	300.7 (11.7)	311.3 (4.44)	259.0^c^
12.0	235.5 (5.72)	343.9 (6.68)	418.3 (19.8)	442.8 (4.09)	360.1^d^
12.5	296.2 (6.80)	442.7 (7.82)	529.0 (11.9)	586.3 (2.54)	463.5^e^
13.0	364.4 (4.10)	547.8 (6.46)	650.5 (22.1)	737.6 (18.1)	575.1^f^
Mean	207.84^a^	307.62^b^	368.54^c^	399.82^d^	–

Different superscript letters (a,b,c, etc.) indicate significant differences among groups using the Duncan Multiple-Range Test (*P* < 0.05).

When comparing with the previous studies, Budiman et al.^28^ produced HHO gas by dry cell and used NaCl as an electrolyte (500 cm^3^ of water + 100 g of NaCl), and they reported that the highest production of HHO gas was 175 cm^3^ at time of (3.0–3.5 min) using battery source (12 V–10 A). Meanwhile, Essuman et al.^4^ observed that increasing electrolyte strength and voltage correspondingly improved the yield of HHO gas. Their findings revealed that the optimal yield rate of 136.2 cm^3^ min^−1^ of HHO gas was achieved when the generator was run at 13 V and 0.025 M KOH. Whereas, Sudrajat et al.^32^ discovered that using a 22.4 g L^−1^ of KOH catalyst resulted in an average HHO gas production of 230.3 cm^3^ min^−1^ at 12 V. Also, they found that the volumes of HHO gas produced were 152, 176, and 258 cm^3^ for HHO gas generator configurations 3A 3C 12N, 3A 3C 16N, and 3A 3C 20N respectively at using 0.02 M KOH for 50 s.

Duncan Multiple Range Test (DMRT) in Table 3 revealed that the mean impact of applied voltage on the HHO production rate, the average values of production rate increased significantly at 5% level (*P* < 0.05) from 99.17 to 575.1 cm^3^ min^−1^ with increased the applied voltage from 10.5 to 13 V.

From Table 3, the results of Duncan’s test for the mean impact of KOH concentration on the rate of HHO gas production rate revealed that the average values of production rate increased significantly from 207.84 to 399.82 cm^3^ min^−1^ with increasing the KOH concentration from 0.05 to 0.20 M.

### Power consumption

**Fig. 5:**
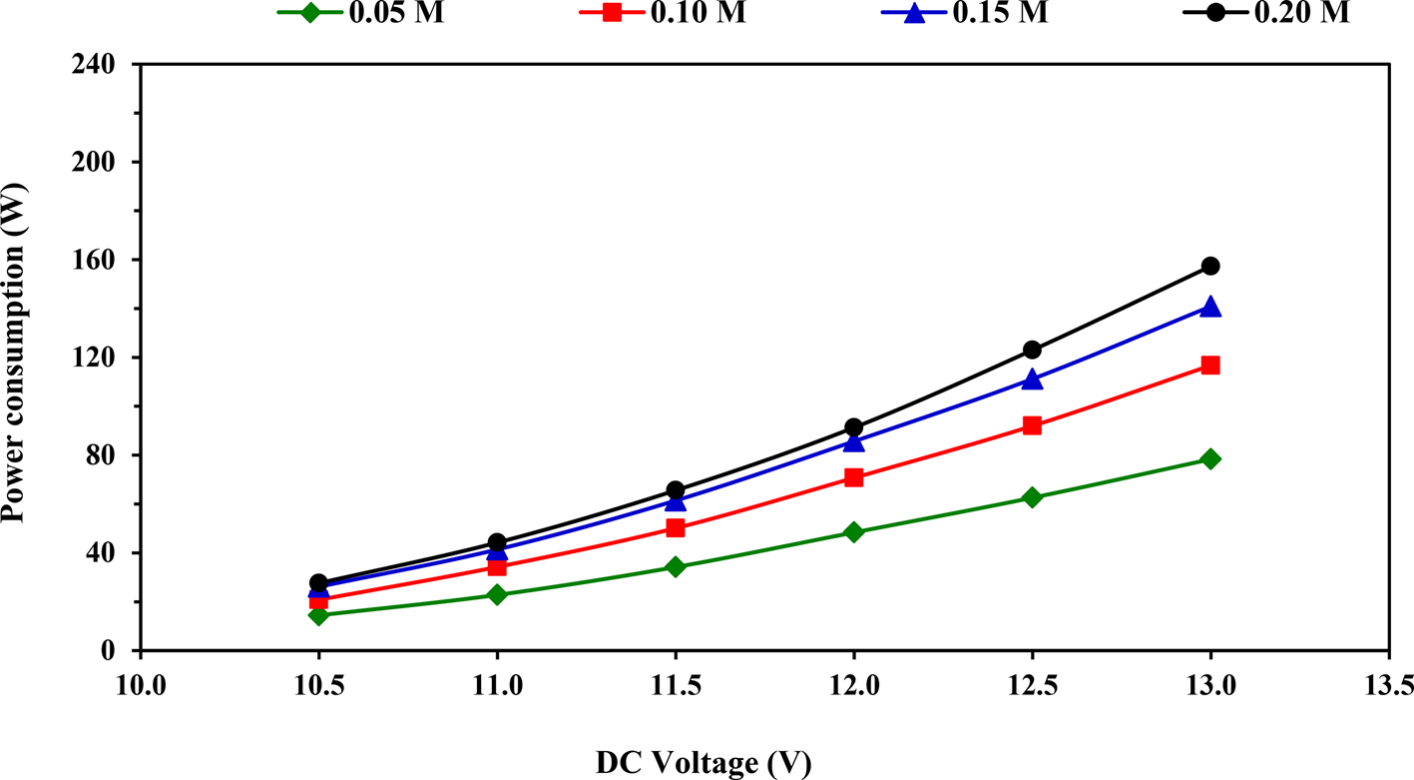
Impact of applied voltage on power consumption at tested KOH concentrations.

Figure 5 illustrates the impact of applied voltage on the consumed power at tested KOH concentrations. It was found that the consumed power increased gradually by increasing the applied voltage for all tested KOH concentrations. This result may be because of the increase in the consumed current with increasing the applied voltage, which followed an increase in power consumption. In addition, increasing the KOH concentration of the electrolyte solution leads to increased electrical conductivity^33^.

The results indicated that the power consumption increased from 14.46 to 78.34, 20.81 to 116.7, 26.16 to 140.9, and 27.62 to 157.3 W with increasing the applied voltage from 10.5 to 13.0 V at four tested KOH concentrations 0.05, 0.10, 0.15 and 0.20 M, respectively as displayed in Table 4. Duncan’s test in Table 4 revealed that the mean impact of applied voltage on the power consumption, the average values of power consumption increased significantly (*P* < 0.05) from 22.26 to 123.3 W with increased the applied voltage from 10.5 to 13 V. Also, the result of Duncan’s test for the mean effect of KOH concentration on power consumption showed that the average values of power consumption increased significantly from 43.47 to 84.85 W with increasing the KOH concentration from 0.05 to 0.20 M.

**Table 4:** Average values of DC power consumption at tested voltages and electrolytic concentrations, [mean value ± (SD)] and Duncan’s test result.

DC voltage (V)	DC Power consumption (W)				Mean
	Electrolytic solution concentration (M)				
	0.05	0.10	0.15	0.20	
10.5	14.46 (0.59)	20.81 (0.47)	26.16 (0.89)	27.62 (1.18)	22.26^a^
11.0	22.81 (0.29)	34.26 (0.34)	41.41 (0.23)	44.24 (0.92)	35.68^b^
11.5	34.21 (0.21)	50.14 (1.31)	61.44 (2.04)	65.60 (0.53)	52.85^c^
12.0	48.38 (0.32)	70.72 (0.63)	85.68 (3.16)	91.31 (1.07)	74.02^d^
12.5	62.59 (0.15)	91.97 (0.92)	111.2 (3.39)	123.0 (3.68)	97.19^e^
13.0	78.34 (0.32)	116.7 (0.86)	140.9 (4.54)	157.3 (5.10)	123.3^f^
Mean	43.47^a^	64.10^b^	77.80^c^	84.85^d^	–

Different superscript letters (a,b,c, etc.) indicate significant differences among groups using the Duncan Multiple-Range Test (*P* < 0.05).

### Change in temperature

**Fig. 6:**
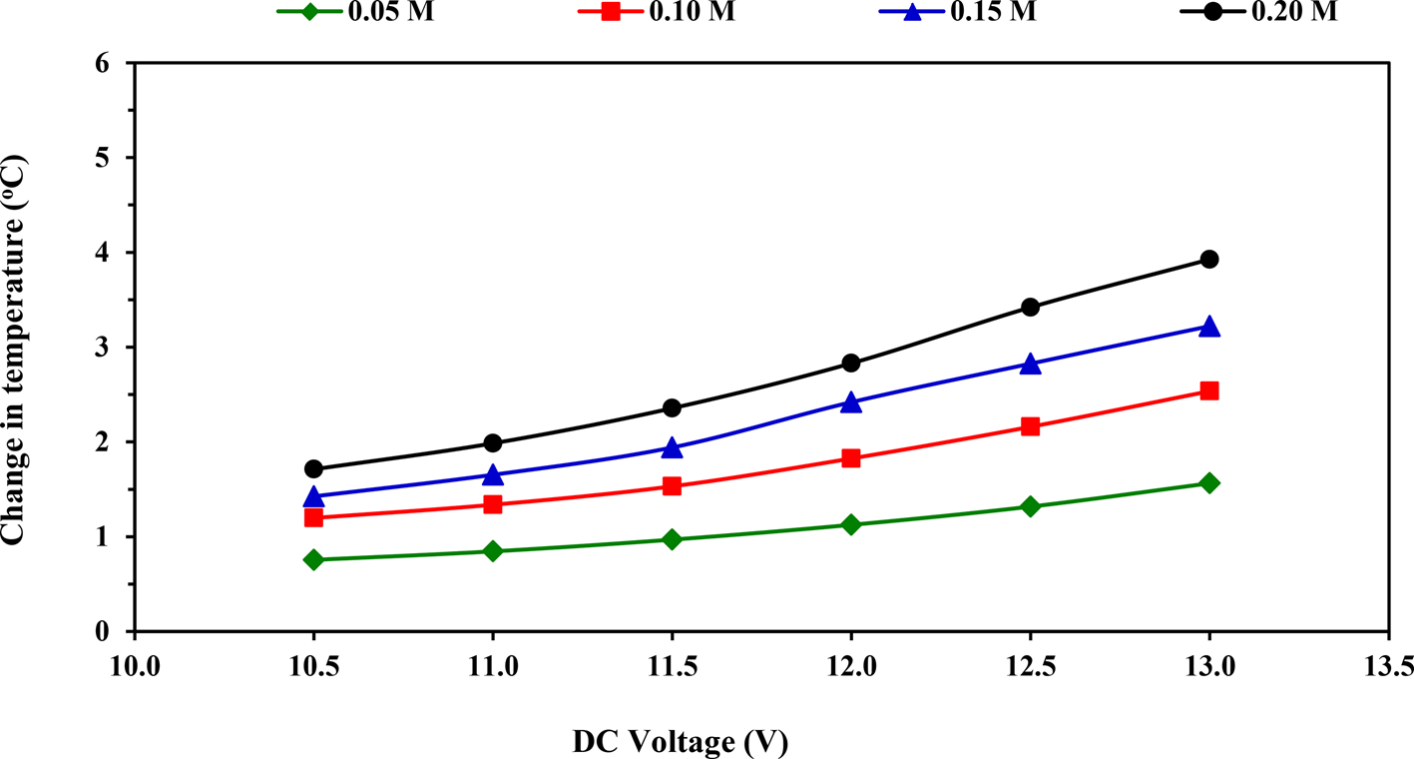
Impact of applied voltage on change in temperature at tested KOH concentrations.

Figure 6 shows the impact of applied voltage on change in electrolyte solution temperature when the experiment is over at tested KOH concentrations. It’s noted that the temperature change increased gradually with increasing the applied voltage for all tested KOH concentrations. Increasing the applied voltage led to an increase in the consumed current and, therefore, followed a rise in power consumption, which increased the HHO production rate. However, there is a negative side effect: an increase in the temperature of electrolyte solution during the electrolysis process^34^. This may occur due to an increase in electron mobility and an increase in heat transfer rate on the plates^16^.

Also, the obtained results revealed that the amount of change in temperature increased from 0.76 to 1.56, 1.20 to 2.54, 1.43 to 3.22, and 1.71 to 3.93 °C with increasing the voltage from 10.5 to 13.0 V at four tested KOH concentrations 0.05, 0.10, 0.15 and 0.20 M, respectively as displayed in Table 5. Duncan’s test result in Table 5 indicates the average impact of applied voltage on the change in electrolyte solution temperature; the average values of change in temperature increased significantly from 1.27 to 2.81 °C with increasing the applied voltage from 10.5 to 13 V. Table 5 also illustrates Duncan’s test result for the average impact of KOH concentration on temperature change. The average temperature change values increased significantly from 1.10 to 2.71 °C with increasing the KOH concentration from 0.05 to 0.20 M.

**Table 5:** Average values of change in temperature at tested voltages and electrolytic concentrations, [mean value ± (SD)] and Duncan’s test result.

DC voltage (V)	Change in temperature (^o^C)				Mean
	Electrolytic solution concentration (M)				
	0.05	0.10	0.15	0.20	
10.5	0.76 (0.05)	1.20 (0.12)	1.43 (0.03)	1.71 (0.05)	1.27^a^
11.0	0.85 (0.03)	1.34 (0.03)	1.65 (0.03)	1.99 (0.03)	1.46^b^
11.5	0.97 (0.03)	1.53 (0.03)	1.94 (0.07)	2.36 (0.03)	1.70^c^
12.0	1.13 (0.01)	1.83 (0.05)	2.42 (0.16)	2.83 (0.06)	2.05^d^
12.5	1.32 (0.04)	2.16 (0.06)	2.83 (0.15)	3.42 (0.11)	2.43^e^
13.0	1.56 (0.09)	2.54 (0.07)	3.22 (0.29)	3.93 (0.19)	2.81^f^
Mean	1.10^a^	1.77^b^	2.25^c^	2.71^d^	–

Different superscript letters (a,b,c, etc.) indicate significant differences among groups using the Duncan Multiple-Range Test (*P* < 0.05).

### Specific energy requirement

**Fig. 7:**
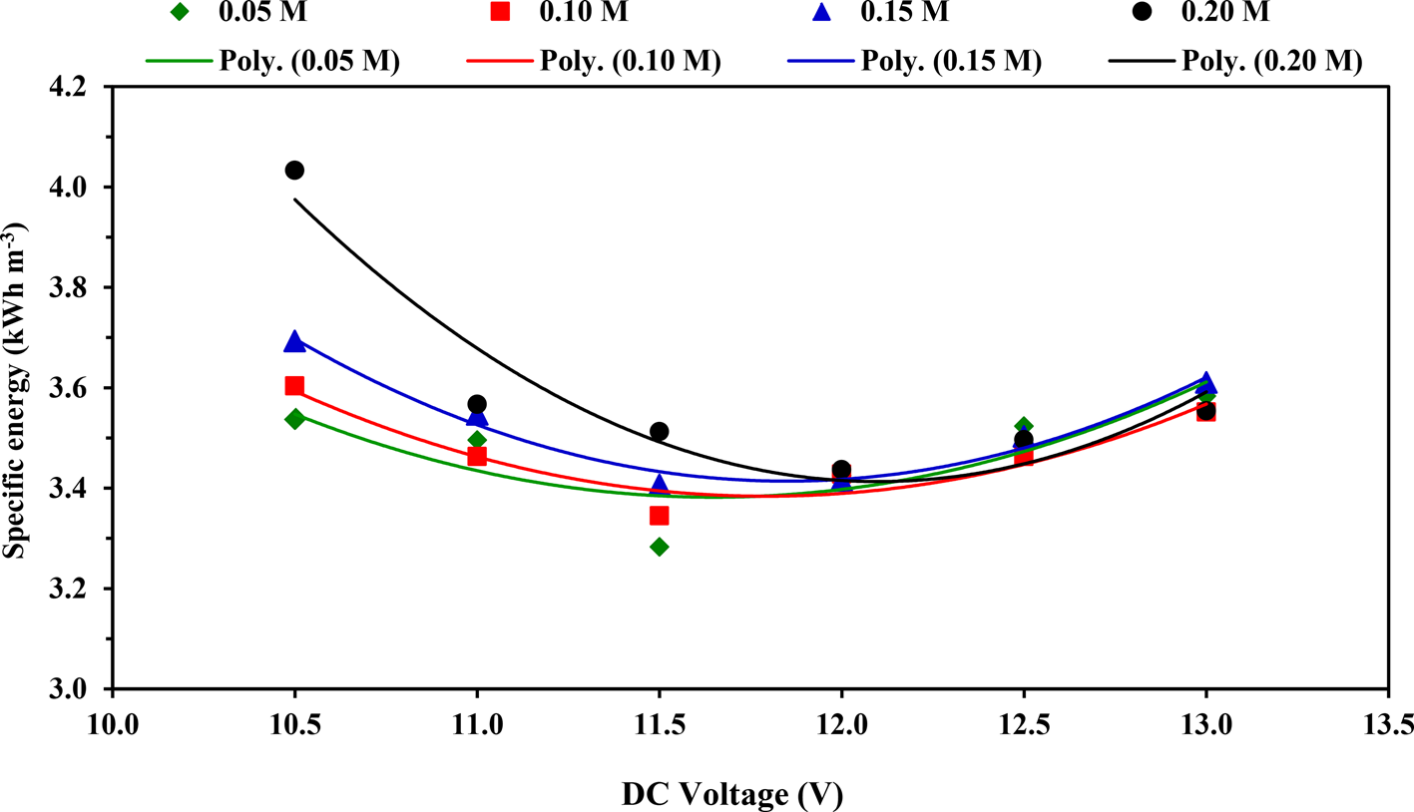
Impact of applied voltage on specific energy at tested KOH concentrations.

Figure 7 shows the impact of applied voltage on the specific energy requirement at tested KOH concentrations. Generally, we discovered that increasing the electrolytic solution concentration from 0.05 to 0.2 M increased the specific energy required for all applied voltages from 10.5 to 13 V. This could be attributed to an increase in the temperature of the electrolytic solution during operation, which is likely to increase current consumption hence increasing the specific energy required for the production of HHO gas according to Nabil^27^ and Nabil and Dawood^7^.

For tested KOH concentrations 0.05, 0.10, and 0.15 M, the results indicated that specific energy values decreased gradually with increasing the applied voltage from 10.5 to 11.5 V. In contrast, as the applied voltage increased from 11.5 to 13 V, the specific energy increased. In comparison, using the KOH concentration of 0.20 M, the specific energy decreased gradually with the increase in the applied voltage from 10.5 to 12.0 V, which increased with the increase in the applied voltage to 13.0 V, as shown in Fig. 7 and Table 6. So, the production of HHO gas is more efficient when the specific energy value drops and less efficient when the specific energy value rises. This could be due to a gradual increase in the electrolyte solution’s temperature resulting in an increase in electric current drawn, which increases power consumption faster than the rate of increased HHO gas production because a part of the electric power is consumed in electrolyte solution heating with agree with Wu et al.^35^ and Hassan et al.^34^. According to the literature, Rusdianasari et al.^36^ employed an HHO gas generator. They found that the specific energy consumption was 29.18 kWh m^−3^. Meanwhile, Patil et al.^37^ found that producing 1 m^3^ of HHO gas needs 15 kWh. Whereas Wu et al.^35^ found that the required specific energy was 3.31 kWh for every 1 m^3^ HHO gas.

**Table 6:** Average values of specific energy at applied voltages and electrolytic concentrations, [mean value ± (SD)] and Duncan’s test result.

DC voltage (V)	Specific energy (kWh m^−3^)				Mean
	Electrolytic solution concentration (M)				
	0.05	0.10	0.15	0.20	
10.5	3.54 (0.16)	3.60 (0.04)	3.69 (0.14)	4.03 (0.05)	3.72^d^
11.0	3.50 (0.08)	3.46 (0.06)	3.55 (0.07)	3.57 (0.07)	3.52^b^
11.5	3.28 (0.16)	3.34 (0.15)	3.41 (0.06)	3.51 (0.06)	3.39^a^
12.0	3.43 (0.08)	3.43 (0.04)	3.42 (0.04)	3.44 (0.04)	3.43^a^
12.5	3.52 (0.08)	3.46 (0.03)	3.50 (0.05)	3.50 (0.10)	3.50^b^
13.0	3.58 (0.04)	3.55 (0.05)	3.61 (0.01)	3.55 (0.03)	3.57^c^
Mean	3.47^a^	3.48^a^	3.53^b^	3.60^c^	–

Different superscript letters (a,b,c, etc.) indicate significant differences among groups using the Duncan Multiple-Range Test (*P* < 0.05).

Duncan’s test in Table 6 revealed that the mean effect of applied voltage on the specific energy requirement, the average values of specific energy decreased significantly (*P* < 0.05) from 3.72 to 3.39 kWh m^−3^ with increasing the voltage from 10.5 to 11.5 V while, the average values of specific energy are not significantly at using voltage from 11.5 to 12.0 V whereas, the average values of specific energy increased significantly from 3.43 to 3.57 kWh m^−3^ with increasing the voltage from 12.0 to 13.0 V.

Also, Table 6 illustrates the result of Duncan’s test for the average impact of KOH concentration on the specific energy requirement; the average values of specific energy increased significantly from 3.48 to 3.60 kWh m^−3^ with increasing the KOH concentration from 0.01 to 0.20 M whereas, the average values of specific energy are not significantly at using KOH concentrations of 0.05 and 0.01 M.

The outcomes also showed that the relationship between specific energy and applied voltage was a second-degree polynomial equation, which can be expressed using Eqs. (6–9) for the tested electrolyte concentrations.

6 $$E_{{s\left( {0.05M} \right)}} = 0.1255V^{2} - 2.9243V + 20.413 \left( {R^{2} = 0.6842} \right)$$

7 $$E_{{s\left( {0.10M} \right)}} = 0.1256V^{2} - 2.9613V + 20.842 \left( {R^{2} = 0.8907} \right)$$

8 $$E_{{s\left( {0.15M} \right)}} = 0.1558V^{2} - 3.6918V + 25.285 \left( {R^{2} = 0.9715} \right)$$

9 $$E_{{s\left( {0.20M} \right)}} = 0.2197V^{2} - 5.3155V + 35.569 \left( {R^{2} = 0.9132} \right)$$

### Efficiency

**Fig. 8:**
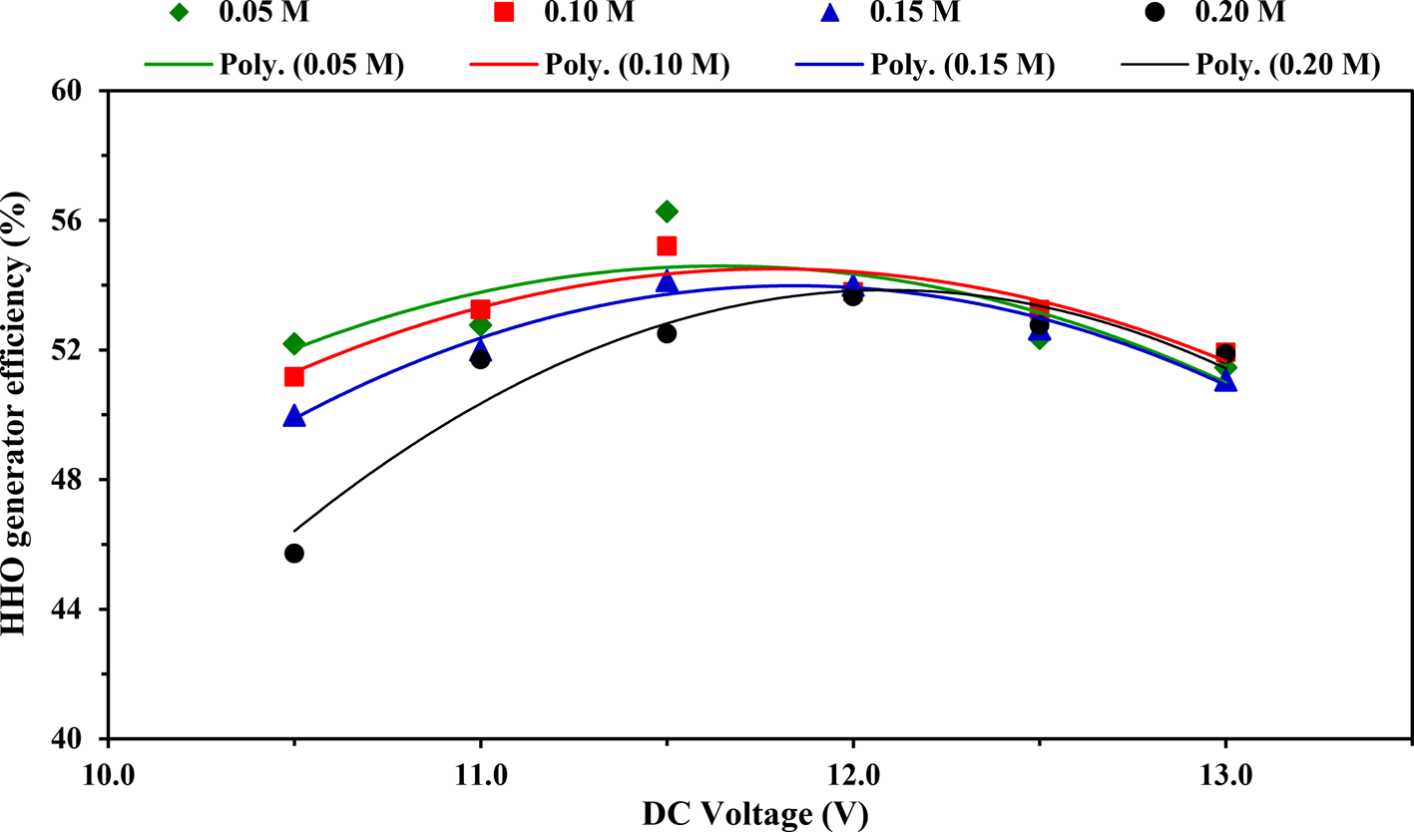
Impact of applied voltage on generator efficiency at tested KOH concentrations.

Figure 8 illustrates the impact of applied voltage on the efficiency of the HHO gas generator at tested KOH concentrations. For tested KOH concentrations 0.05, 0.10, and 0.15 M, the results indicated that the values of HHO gas generator efficiency increased gradually with increasing the applied voltage from 10.5 to 11.5 V, after which it decreased with increasing the applied voltage up to 13.0 V. In comparison, when using the KOH concentration of 0.20 M, the generator efficiency increased gradually with increasing the applied voltage from 10.5 to 12.0 V, after which it decreased with increasing the applied voltage up to 13.0 V, as shown in Fig. 8 and Table 7, a similar trend of this outcome was reported El Kady et al.^2^. The decline in HHO gas generator efficiency could be attributed to loss some of the consumption electrical power in raising the temperature of the electrolyte solution which causes decreasing of HHO production rate^7,16^.

**Table 7:** Average values of HHO gas generator efficiency at tested voltages and electrolytic concentrations, [mean value ± (SD)] and Duncan’s test result.

DC voltage (V)	HHO gas Generator efficiency (%)				Mean
	Electrolytic solution concentration (M)				
	0.05	0.10	0.15	0.20	
10.5	52.18 (2.33)	51.16 (0.54)	49.98 (1.85)	45.72 (0.62)	49.76^d^
11.0	52.76 (1.16)	53.24 (0.85)	52.00 (1.09)	51.70 (1.04)	52.42^b^
11.5	56.27 (2.70)	55.20 (2.38)	54.13 (0.91)	52.49 (0.85)	54.52^a^
12.0	53.84 (1.25)	53.79 (0.66)	53.99 (0.65)	53.65 (0.61)	53.82^a^
12.5	52.35 (1.24)	53.24 (0.50)	52.64 (0.77)	52.76 (1.47)	52.75^b^
13.0	51.45 (0.51)	51.92 (0.70)	51.06 (0.16)	51.87 (0.47)	51.58^c^
Mean	53.14^a^	53.09^a^	52.30^b^	51.37^c^	–

Different superscript letters (a,b,c, etc.) indicate significant differences among groups using the Duncan Multiple-Range Test (*P* < 0.05).

Duncan’s test in Table 7 indicates the average impact of applied voltage on the efficiency of HHO generator. The average values of efficiency of HHO gas generator increased significantly from 49.76 to 54.52% with increasing the applied voltage from 10.5 to 11.5 V. In contrast, the average HHO generator efficiency values are not significant at applied voltage from 11.5 to 12.0 V. In contrast, the average values of efficiency of HHO generator decreased significantly from 53.82 to 51.58% with increasing the applied voltage from 12.0 to 13.0 V.

Also, Table 7 illustrates the Duncan’s test result for the mean effect of KOH concentration on the efficiency of HHO generator. The average values of generator efficiency decreased significantly from 53.09 to 51.37% with the increase in the KOH concentration from 0.01 to 0.20 M. Meanwhile, the average values of generator efficiency are not significant when using KOH concentrations of 0.05 and 0.01 M.

In comparison with previous studies, Sudarmanta et al.^25^ stated that the HHO gas generator efficiency was 20.06% using a PWM system with a 40% duty cycle, a specific energy input of 33,121 MJ kg^−1^, and generator temperature can be maintained below 60 °C. While, Najafi et al.^30^ demonstrated that the generator efficiency of HHO gas improved with increasing the concentration of NaOH catalyst from 2 to 6 g L^−1^ and then dropped with increasing the catalyst concentration from 6 to 10 g L^−1^; also, they found that the maximum efficiency of 39.12% was achieved at 6 g L^−1^ of NaOH catalyst.

Moreover, the findings revealed that the relationship between HHO generator efficiency and applied voltage was a second-degree polynomial equation, which can be expressed for the tested electrolyte concentrations using Eqs. (10–13).

10 $$\eta_{{s\left( {0.05M} \right)}} = - 1.9544V^{2} + 45.511V - 210.36 \left( {R^{2} = 0.654} \right)$$

11 $$\eta_{{s\left( {0.10M} \right)}} = - 1.9306V^{2} + 45.503V - 213.62 \left( {R^{2} = 0.871} \right)$$

12 $$\eta_{{s\left( {0.15M} \right)}} = - 2.2806V^{2} + 54.006V - 265.75 \left( {R^{2} = 0.965} \right)$$

13 $$\eta_{{s\left( {0.20M} \right)}} = - 2.9338V^{2} + 70.950V - 375.11 \left( {R^{2} = 0.926} \right)$$

## Conclusion

The developed HHO generator exhibits ease of manufacturing, operation, and maintenance, and its design has successfully facilitated the production of HHO gas. Within the range of tested potassium hydroxide (KOH) concentrations (0.05 to 0.20 M) and applied voltages (10.5 to 13.0 V), the results demonstrated a significant increase in HHO gas production rate with higher applied voltages and electrolyte solution concentrations. It was also observed that the power consumption for HHO gas production increased notably with increasing applied voltage and electrolyte concentration. Furthermore, an increase in applied electrolyte concentration and voltage led to a corresponding rise in temperature. From this study, the optimum conditions for producing HHO gas ranged from 11.5 to 12 V for voltage and from 0.05 to 0.10 M for KOH concentration according to the lowest specific energy and highest HHO gas generator efficiency. Under the previous optimum conditions, the highest productivity, specific energy, and efficiency of the HHO gas generator were 343.9 cm^3^ min^−1^, 3.43 kW h m^−3^, and 53.79%, respectively, using 12.0 V for applied voltage and 0.10 M for electrolyte solution concentration. In the future, other variables and new technologies will be studied to enhance generator efficiency, in addition to using renewable sources (such as solar energy, wind energy, etc.) for producing eco-friendly fuel (hydrogen).

## Data Availability

All data and materials are available with the corresponding author upon request. in this email: khaledabdeen@azhar.edu.eg.
